# Comparative genomics of Vibrio toranzoniae strains

**DOI:** 10.21203/rs.3.rs-4360386/v1

**Published:** 2024-05-20

**Authors:** Rubén Barcia-Cruz, Sabela Balboa, Alberto Lema, Jesús L. Romalde

**Affiliations:** Universidade de Santiago de Compostela; Universidade de Santiago de Compostela; Universidade de Santiago de Compostela; Universidade de Santiago de Compostela

**Keywords:** Vibrio toranzoniae, genome sequencing, phylogenomics, virulence genes

## Abstract

*Vibrio toranzoniae* is a marine bacterium belonging to the Splendidus clade, originally isolated from healthy clams in Galicia (NW Spain). Its isolation from different hosts and seawater indicated two lifestyles and wide geographical distribution. The aim of the present study was to determine the differences at genome level among strains, as well as to determine their phylogeny. For this purpose, whole genomes were sequenced by different technologies and the resulting sequences corrected. Genomes were annotated and compared with different online tools. Furthermore, the study of core and pan genome was examined, and the phylogeny was inferred. The content of the core genome ranged from 2,953 to 2,766 genes and that of the pangenome from 6,278 to 6,132, depending on the tool used. The comparison revealed that although the strains shared certain homology, with DDH values ranging from 77.10 to 82.30 and values of OrthoANI higher than 97%,notable differences were found related to motility, capsule synthesis, iron acquisition system or mobile genetic elements. The phylogenetic analysis of the core genome did not reveal a differentiation of the strains according to their lifestyle, but that of the pangenome pointed out certain geographical isolation in the same growing area. The study led to a reclassification of some isolates formerly described as *V. toranzoniae* and manifested the importance of cured deposited sequences to proper phylogenetic assignment.

## Introduction

*Vibrio toranzoniae* is a marine bacterium of the Splendidus clade belonging to the *Vibrio genus* (Lasa et al., 2013). To date, the Splendidus clade is the largest clade within the genus *Vibrio*, containing 18 species: *V. artabrorum, V. atlanticus, V. celticus, V. chagasii, V. coralliirubri, V. crassostreae, V. cyclitrophicus, V. echinoideorum, V. fortis, V. gallaecicus, V. gigantis, V. kanaloae, V. lentus, V. pelagius, V. pomeroyi, V. splendidus, V. tasmaniensis,* and *V. toranzoniae* (Pérez-Cataluña et al., 2016; Poli et al., 2018; Hira et al., 2019; Jiang et al., 2022). Another species, ‘*V. profundi*’ has been described as belonging to this clade, but has not been validated yet (Zhang et al., 2019). The Spendidus clade comprises several pathogenic species, such as *V. crassostreae* (Bruto et al., 2017), *V. tasmaniensis* (Duperthuy et al., 2011; Rubio et al., 2019) or *V. splendidus* (Thomson et al., 2005), which can cause considerable losses in the aquaculture industry (Dubert et al., 2017).

*Vibrio toranzoniae* was first isolated from clams (*Ruditapes phillipinarum* and *R. decussatus*), during a study of microbiota associated with reared healthy clams in Galicia, Spain (Lasa et al., 2013). The subsequent isolations of the species from seawater in Valencia (Spain) and from seawater and hatchery rearing systems for production of blue mussel (*Mytilus galloprovincialis*) in Australia (Kwan and Bolch, 2015) indicated that the host and geographical distribution of this species was wider than expected. Also, three isolates from moribund red conger eel (*Genypterus chilensis*) in Chile were initially attributed to *V. toranzoniae*, although in this work are being reclassified as *V. kanaloae*.

The genomes of bacterial species give us information essential to elucidate taxonomy in closely related species, but also about properties of interest, such as drug sensitivity or virulence factors. To tackle such information, the genomics revolution that made thousands of prokaryotic genomes available to the scientific community has come hand in hand with a revolution in computational tools to compare these genomes (Setubal et al., 2018). The development of bioinformatics tools and web-based databases provides an online, user-friendly method for the identification and prediction of relevant information from genome data, as antimicrobial resistance (Babiker 2019) or virulence factor genes (Waseem 2017). The resulting differences between genomes of interest may be crucial to decipher the genetic basis of pathogenicity or virulence capacities among strains, being such information of high relevance in tracing mortality outbreaks. In addition, it may give some clues on different survival strategies that vibrios can develop to persist in the environment..

In this work, we use general genomic features, variable characteristics in factors of interest, evidence of genomic exchange, phylogenetic relationships and the study of the core and pan genome to compare a collection of *V. toranzoniae* strains. We provide an example of how comparative genomics can help to unravel the taxonomy of a complicated group and how it can help to obtain information regarding the biology of the group.

## Materials and Methods

### Bacterial Strains

Strains included in the comparison analysis are listed in Table 1. These comprise four motile, facultative anaerobic marine strains isolated from healthy cultured adult clams (*R. philippinarum* and *R. decussatus*) in Galicia (Spain), including the type strain of the species CECT 7225^T^ (= Vb 10.8^T^), together with two environmental strains isolated from seawater in Valencia (Spain) (kindly donated by Prof. M.J. Pujalte). Additionally, the three strains isolated from red conger eel (*Genypterus chilensis*) in Chile (Lasa et al., 2015), were initially added in the comparisons, until the study revealed that they belong to *V. kanaloae* species. Stock cultures of the isolates were stored at −80°C in marine broth supplemented with 20% (v/v) glycerol, and routinely cultured in marine agar plates at 25°C.

### Genomic DNA Extraction, Sequencing, Assembly and Annotation

Genomic DNA was extracted using the QIAamp DNA minikit (Qiagen), following the manufacturer’s protocol. The genomes of *V. toranzoniae* strains were sequenced at David H. Murdock Research Institute (DHMRI) of the University of North Carolina (Kannapolis, North Carolina) using a HiSeq 2500 sequencing technology (Illumina) with 2 × 100-bp paired-end reads, and at FISABIO (Valencia, Spain) using a MiSeq System sequencing technology (Illumina) with 2 × 300-bp paired-end reads. Additionally, the genomes of the type strain CECT 7225^T^ and the environmental isolate 96–376 were sequenced at SNPsaurus at the University of Oregon, using a PacBio technology.

The Illumina reads were analysed for quality control using FASTQC (Brabaham Bioinformatics). Reads were trimmed and filtered to remove adapters and low-quality bases, using Trimmomatic 0.32 (Bolger et al., 2014) program. The remaining reads were used for the genome assembly, performed with the SPAdes 3.6.1 the novo assembler tool (Nurk et al., 2013), and QUAST (Gurevich et al., 2013) software was used to evaluate the assembly.

The whole genomes of the strains were deposited at GenBank under the accession numbers GCA-001541335.1 (*V. toranzoniae* CECT 7225^T^ ), GCA-009906155.1 (*V. toranzoniae* 96–373), GCA-009906235.1 (*V. toranzoniae* 96–376), GCA-009906185.1 (*V. toranzoniae* CMJ 9.4), GCA-009906175.1 (*V. toranzoniae* CMJ 9.11) and GCA-009906085.1 (*V. toranzoniae* Cmf 13.9).

### Genomic Indices

To measure the similarity among the strains, the *in silico* DNA-DNA hybridization (dDDH) and the Orthologous Average Nucleotide Identity (OrthoANI) were calculated between pairs of genomes. dDDH was calculated with GGDC software, using the results offered by formula 2 (Meier-Kolthoff et al., 2013). OrthoANI was calculated using ChunLab’s Orthologous Average Nucleotide Identity Tool (OAT), with an algorithm demarcation cut-off of 95 ~ 96% (Lee et al., 2016).

### Sequence correction

Obtention of long sequencing reads has been associated with low sequencing accuracy. Thus, several approaches have been proposed to enhance the quality of long sequence reads, such as hybrid assemblies, higher sequencing coverage or sequence correction (Mahmoud et al., 2019). In this work, complementation of PacBio low-accuracy long reads with Illumina high-accuracy short reads were performed for both CECT 7225^T^ and 96–376 strains. Therefore, the PacBio sequenced genomes were first assembled with Flye version 2.6 (Kolmogorov et al., 2019). Next, Minimap2 version 2.17 (Li, 2018) was used to mapping back the genomes. Then, PacBio sequences were polished with Racon version 1.4.3 (Vaser et al., 2017). After that, alignment with Illumina sequences was achieved with Bowtie2 version 2.3.5 (Langmead and Salzberg, 2012). Finally, the result was polished with and Pilon version 1.2.3 (Walker et al., 2014) to accomplish the hybrid genome.

### Differential Phenotypical Features

The exploration of genes and systems within the strains was accomplished using different annotation tools, Rapid Annotations using Subsystems Technology (RAST) server (Overbeek et al., 2014), the Annotation Tools of PATRIC 3.5.43 server (Brettin et al., 2015) and PROKKA V1.13.3 (Seemann, 2014).

To corroborate the results observed in the genomic analyses, some biochemical tests were carried out. Capsule production was assessed by culturing the *V. toranzoniae* strains on Congo red agar (CRA) plates as described by Freeman et al. (Freeman et al., 1989). After and incubatation for 48 h at 25 °C, black colonies were considered as capsule producers. Detection of siderophores was assayed culturing the strains on chrome azurol S (CAS) blue agar plates, being orange halos around the colonies indicative of siderophore production (Schwyn and Neilands, 1987; Lynne et al., 2011). Finally, motility was observed by optical microscopy and soft agar. Presence of flagella was determined by specific staining using Leifson dye (Leifson, 1930), and visualizing the preparations in a 100x optical microscope.

### Genomic Exchange

Different online tools were used for the search of genetic transfer. Therefore, antiSMASH 5.0 (Blin et al., 2019) was utilized for finding secondary metabolite clusters, PHASTER (Arndt et al., 2016) to identify prophages sequences, DefenseFinder to detect known anti-phage systems (Abby et al., 2014; Tesson et al., 2022) and Comprehensive Antibiotic Resistance Database (CARD) (Alcock et al., 2019) for the detection of antimicrobial resistance genes, using the Resistance Gene Identifier (RFI) tool. Identification of Genomic Islands was performed with IslandViewer 4 (Bertelli et al., 2017), using IslandPick, SIGI-HMM and IslandPath-DIMOB methods, by comparison with *V. splendidus* LGP32, *V. vulnificus* YJ016 and *V. anguillarum* 775 as the reference genomes. For the search of CRISPR-Cas sequences, genomes were analysed using CRISPRCasFinder online tool (Couvin et al., 2018).

### Phylogenetic Analysis

Core and pan- genome phylogenomic analysis of the species was performed using the three different algorithms of GET_Homologues software (Contreras-Moreira and Vinuesa, 2013), namely bi-directional best-hits (BDBH), Cluster of Orhologous Groups triangle (COGtriangle) and Markov Clustering of Orthologous (OrthoMCL). For the appropiate use of GET_Homologues, functional annotation of genomes was carried out with PROKKA V1.13.3.

Core and pan-genome phylogenomic analyses were also performed using Roary software (Page et al. 2015). Phylogenomic trees were visualized using FigTree version 1.4.3 (Rambaut, A. 2016).

## Results and Discussion

### Reclassification of former V. toranzoniae R17 as V. kanaloae R17

**Figure 1:**
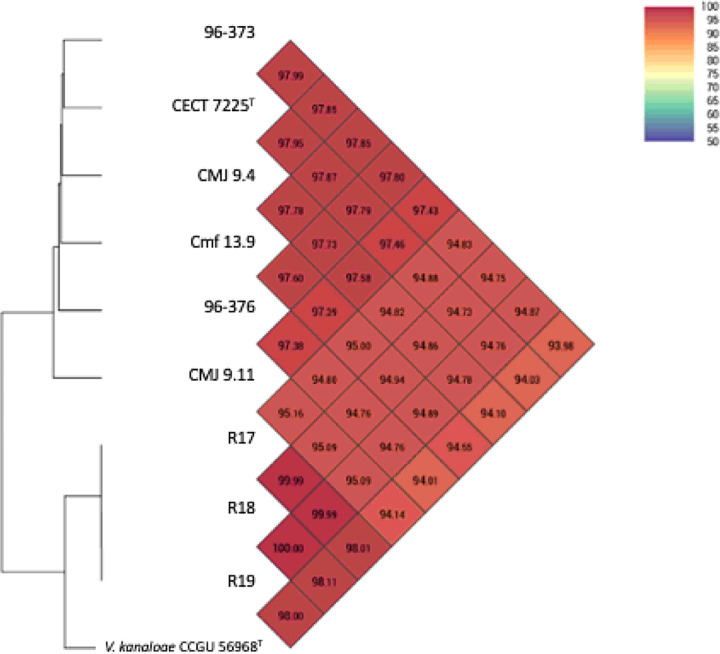
Values of OrthoANI for the strains of study.

**Figure 2:**
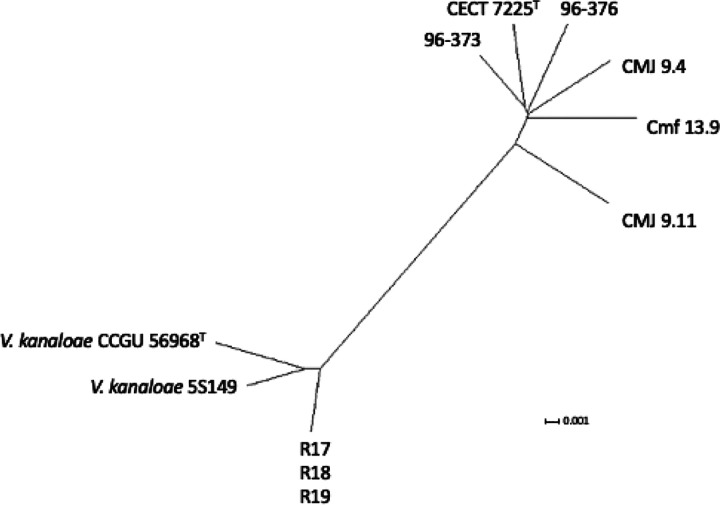
Phylogenetic tree of the core genome of *V. toranzoniae* and *V. kanaloae* strains.

According to genome sequence similarity and genomic indexes, the genomes of *V. toranzoniae* strains separated in two well defined clusters: on the one hand, the six strains isolated from clams and seawater in Europe, and on the other hand, the three strains isolated in Chile together with *V. kanaloae* (strains CCUG 56968^T^ and 5S149)(Tables 2; Figs. 1, 2), a *Vibrio* species that was first isolated from diseased oyster (*Ostrea edulis*) larvae in France (Thompson et al., 2003a). Our results confirmed also that the three Chilean isolates are clones, with a dDDH value of 100%, and an OrthoANI value of 99.99–100% (Table 2, Fig. 1). Besides, OrthoANI and dDDH results showed that the chilean isolates are in fact *V. kanaloae*. OrthoANI and dDDH values between these isolates and *V. toranzoniae* strains were below the cut-off values proposed for the delineation of new species (< 96% and < 70%, respectively) (Konstantinidis and Tiedje, 2005; Goris et al., 2007). On the contrary, values for these genomic indexes compared to type strain *V. kanaloae* CCUG 56968^T^, were higher than 98.0% and 86%, respectively. Accordingly, the core-genome -based phylogenetic tree (Fig. 2) reinforced the existence of two separate monophyletic branches. Thus, based on these results, we proposed the assignation of Chilean isolates to *V. kanaloae*.

Revising the genetic sequences available at NCBI, we discovered that one of the two sequences deposited as the 16S rRNA gene of *V. kanaloae* type strain LMG 20539^T^ was poorly named. Therefore, the 16S rRNA gene sequence with accession number AJ316193 (Thompson et al., 2001) coincided with *V. kanaloae* with 100% of similarity, followed by *V. toranzoniae* with 99.66%. Conversely, the other 16S rRNA gene sequence available, with accession number AM162657 (deposited by Le Chevalier, P. et al., unpublished), corresponded to *V. atlanticus* (99.93% of similarity), followed by *V. tasmaniensis* (99.86%), *V. lentus* (99.78%) and then *V. toranzoniae* (98.78%) and *V. kanaloae* (98.77%).

The wrong sequence AM162657 was deposited in 2005, when the second most similar species *V. tasmaniensis* had been already described (Thompson et al., 2003b). In addition, an identical sequence to AM162657 was submitted in 2011, with accession number NR_042468 and processed by NCBI staff, when both *V. tasmaniensis* and *V. atlanticus* 16S rRNA gene sequences were available. For the latter, 16S rRNA gene sequence was deposited in 2007, with accession number EF599163 (Beaz-Hidalgo et al., 2008). This last mislabelled sequence (AM162657) was uploaded by the National Center for Biotechnology Information for its NCBI RefSeq Targeted Loci Project, which includes curated RefSeq records and selected validated GenBank sequences for curated BLAST databases.

It has been highlighted previously that sequences wrongly deposited as type strains may lead to errors in further studies that depend on public databases. That was the case for the so-called *Lelliottia nimipressuralis* type strain SGAir0187 (Heinle et al., 2018), that was misclassified due to a false type strain and was not a strain of the species (Salvà-Serra et al., 2019). Also, Beaz-Hidalgo and coworkers (2015) detected at least 12 misidentified *Aeromonas* genomes among the 44 deposited at the NCBI, insisting these authors in the need of measures to prevent this kind of chaining errors.

In our case, the bad deposit of sequences led to the misassociation of the Chilean isolates with *V. toranzoniae* rather than *V. kanaloae* (Lasa et al., 2015). Considering all the results together and to avoid future problems, we have updated the taxonomic assignation of strain R17 and its deposited sequence (accession number GCA-001995825.2) to *V. kanaloae*.

### Genomic Indices

The genome size of the *V. toranzoniae* strains studied ranged from 4.3 to 4.7 Mb, being 4.5 Mb the average size of the species (Table 3). This genome size is in accordance with the expected for a species of *Vibrio* genus (Thompson et al., 2009). A minimum of 3,826 and maximum of 5,184 coding sequences were predicted using RAST annotation server for the different strains. For RNAs amount, the number oscillated between 126 and 188.

G + C content was practically the same among the strains, varying from 43.8 to 44 mol%, in the range for *Vibrio* species (Table 3). Values of OrthoANI among *V. toranzoniae* ranged from 94.73 to 100% (Fig. 1) and dDDH oscillated between 58.50 and 100% (Table 2).

Although several studies reported that genome size and G + C content show a correlation with the ecological strategies of marine bacteria (Giovannoni et al., 2014; Luo and Moran, 2015), our results did not show differences between the free-living bacteria (*V. toranzoniae* 96–373 and 96–376) and those associated to a host (*V. toranzoniae* CECT 7225T, CMJ 9.4, CMJ 9.11, Cmf 13.9).

### Complete genome sequencing of type strain Vibrio toranzoniae CECT 7225^T^

*V. toranzoniae* was first described based on four isolates from cultured clams in Galicia (NW Spain), designating the strain CECT 7225^T^ as the type strain of the species (Lasa et al., 2013, 2016). For this strain, the read depth obtained sequencing by PacBio technology was 92x, determining the genome size in 4,605,941 bp assembled in two contigs, consistently with the possession of two chromosomes by many species of the *Vibrio* genus, one larger and one smaller of approximately 3.2 and 1.4 Mb, respectively. G + C content was 44 mol% and no plasmids were identified.

The complementation between short Illumina and long PacBio reads did not significantly improved the genome assembly compared to PacBio-only sequencing, since the corrected genome size was 4,605,997 bp, only 56 bp longer than Pac-Bio-only sequenced genome.

With respect to strain 96–376, we were unable to close the genome, and the complementation between Illumina and PacBio reads yielded 6 contigs with a total genome size of 4,370,366 bp, that is, 31,016 bp less than PacBio assembly.

### Core-genome analysis of V. toranzoniae

**Figure 3:**
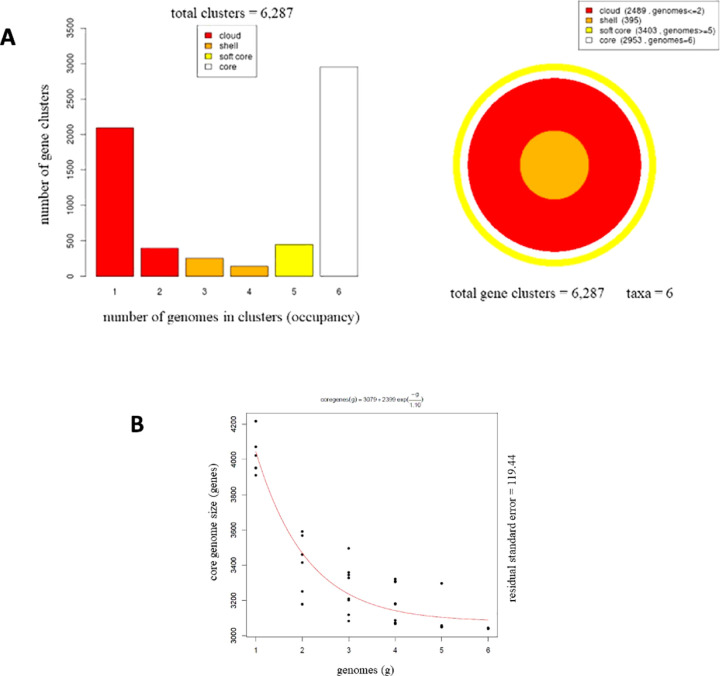
Pangenome analysis of *V. toranzoniae* strains. (A) Partition of the OMCL pangenomic matrix into shell, cloud, soft-core, and core compartments; (B) Estimate size of core genome.

**Figure 4:**
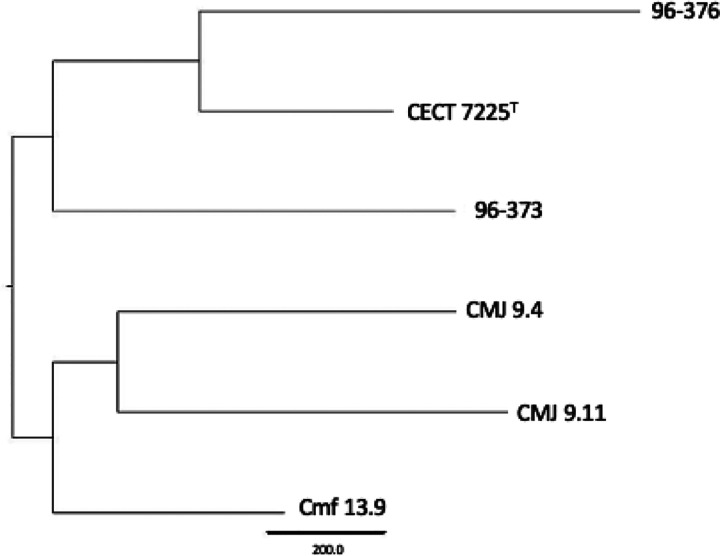
Phylogenomic tree based on pangenomic matrix of *V. toranzoniae*strains.

Core-genome analysis of the *V. toranzoniae* strains with GET_Homologues revealed a pan genome of 6,287 gene clusters, that is, shared by the six isolates included in the study. Of these 6,287 genes, 3,404 were shared by 5 isolates or more (soft core), 2,489 genes were only present in 2 or less taxa (cloud genome), 395 genes were remaining genes shared by several taxa (shell genome) and 2,953 genes were showed by all strains studied (core genome) (Fig. 3A). As seen in Fig. 3B, core genome moderately decreases when more genomes are included, whilst the pan genome acts in reverse. Phylogenomic tree based on pangenomic matrix of *V. toranzoniae* strains is represented in Fig. 4. Using Roary software, from the total pan genome of 6,132 genes, 2,766 belonged to the core genome (shared by 99–100% of taxa), whereas 3,366 formed the shell genome (shared by 15–95% of taxa).

The phylogenomic analysis of the core genome (Fig. 2) did not reveal a differentiation between strains according to the lifestyle either. However, when looking at the phylogenomic tree (Fig. 4), we observed a site-specific differentiation of the three strains isolated from clams in Camariñas (Galicia, Spain), thus sharing the same growing area. Since the pangenome comprises more genes, including those not shared by all strains, this could indicate a local episode of horizontal gene transfer. Consequently, geographical conditions appear to be more decisive than lifestyle or host in *V. toranzoniae* strains. Further studies are needed to con rm such hypothesis.

### Genomic Features

Despite it was not observed a phylogenetic divergence between strains with different lifestyles, some notable differences in gene content were observed.

All strains except one, the environmental strain 96–376, showed the presence of the genes related to flagellar synthesis and regulation. Absence of motility in 96–376 strain was similarly observed in soft agar and checked by optical microscopy. The rest of strains exhibited motility in both soft agar and optical microscopy, being stained flagella observed in bacterial preparations at 100x optical microscopy (data not shown).

Likewise, the environmental strain 96–376, together with the other strain isolated from seawater 96–373, did not exhibit the genes for the rhamnose synthesis pathway, involved in the synthesis of the capsule. This biosynthetic pathway is common and highly preserved across both Gram-positive and Gram-negative bacteria, involving four distinct enzymes that transform glucose into dTDP-L-rhamnose. The initial enzyme in this pathway, glucose-1-phosphate thymidylyltransferase, is responsible for attaching a thymidylmonophosphate nucleotide to Glu-1-P. The resulting dTDP-glucose is further oxidated an d dehydrated by the enzyme dTDP-d-glucose 4,6-dehydratase. Subsequently, a third enzyme, dTDP-6-deoxy-d-xylo-4-hexulose 3,5-epimerase, facilitates a double epimerisation at the C3 and C5 positions. In the nal step, the dTDP-6-deoxy-l-lyxo-4-hexulose reductase reduces the C4 keto group to produce the final product, dTDP-l-rhamnose. On the other hand, the type strain CECT 7225^T^ lacked the reductase gene in this dTDP-rhamnose pathway, and the strain Cmf 13.9 was the only one hosting the thymidylyltransferase.

Presence of capsule was also assessed by growing in CRA plates. After 48 h of incubation, all the strains presented black colonies indicating the production of capsule although, according to the absence of rhamnose-synthesis pathway, strains 96–373 and 96–376 showed the lowest production, indicating also that rhamnose is important but not exclusive for capsule production. The presence of capsule in all the strains could be explained by the advantages that the extracellular polysaccharides confer for environmental survival, but also for host invasion, colonization, persistence and eventually pathogenesis (Bian et al., 2021). Contrary to what it was initially thought, capsule provide protection from physical and chemical stresses without detriment of a high transference of genetic materials between bacteria (Rendueles et al., 2018).

All the strains showed the presence of genes for the transport of iron and for the siderophore aerobactin, despite aerobactin synthase protein IucC was only present in strain 96–373. Nevertheless, only the strain Cmf 13.9 contained the kit of genes for the siderophore assembly. According to this, Cmf 13.9 was the only isolate capable to form orange halo around blue around the colonies in CAS plates, which is indicative of siderophore production.

Related to virulence factors (Table 4), all the strains hosted a vibriolysin and a haemolysin, putative for the case of CMJ 9.11. Besides, all the strains exhibited T1SS secreted agglutinin RTX toxin proteins, witj the exception of the type strain CECT 7225^T^. The strains also manifested the presence of the related Ca^2+^ binding proteins and the type I secretion system, and components necessary for the extracellular secretion, such as a TolC outer membrane protein, an ATP-binding cassette (ABC) and a LapC membrane fusion. Despite the presence of vibriolysins and haemolysins, which in other vibrios have been described as virulence factors (Yuan et al., 2020; Galvis et al., 2021), none of the strains of *V. toranzoniae* cause mortality for clam or turbot (data not shown). This led us to speculate that vibriolysins might not be expressed or that some of the regulation factors are absent. These observations suggest that the *V. kanaloae* strain R17 (reclassified in this work) isolated from moribund red conger eel in Chile could have been the responsible etiological agent, so that *V. toranzoniae* would remain only as a potential pathogen.

Genomic differences between closely related strains are usually concentrated in strain-specific regions of the chromosomes known as genomic islands, that are generally acquired by HGT and that contain adaptive traits that can be linked to niche adaptation (Dobrindt 2004, Penn 2009). Using IslandViewer 4, Genomic Islands (GIs) were identified by SIGI-HMM and Island-Path-DIMOB methods, but not by IslandPick method (Table 5). For all the strains, the highest number of GIs was found by the SIGI-HMM method. The strain showing the highest GIs number was CMJ 9.4. Mobile elements, phage proteins, glycosyltransferases, lipid metabolism proteins and hypothetical proteins were the most found proteins within identified GIs. Iron acquisition system proteins, L-ectoine synthase or MSHA pilin proteins were also found.

### Horizontal Gene Transfer evidences

The evidenced high gene transfer was assessed by different indicators. For example, the abundance of secondary metabolites, which is indicative of genomic exchange since many of them are acquired by horizontal gene transfer (Khaldi et al., 2008). A total of six secondary metabolites were identified using AntiSMASH (Table 4). From them, five were distributed in all strains (polyunsaturated fatty-acid (PUFA) cluster, ectoine, bacteriocin, arylpolyene and betalactone). These secondary metabolites are related with the adaptation of the bacteria to marine environment (Jensen 1996, de Carvalho 2010), found in different marine bacterial genus. Thus, PUFAs are produced by different marine bacteria such as *Vibrio, Photobacterium, Psychromonas or Shewanella*, enabling the transportation of nutrients through the membrane and maintaining its fluidity in the deep-sea cold environment in which these genera inhabit (Moi et al., 2018). Aryl polyenes were described as natural bacterial products which protect bacteria from reactive oxygen species (Schöner et al., 2016). Also, bacteriocin and betalactone are compounds produced by bacteria which show inhibitory or killing activities against other cells (Manivasagan et al., 2014; Yang et al., 2014). Finally, ectoine is an organic compound whose accumulation within the cell allows bacteria to keep turgor pressure under high osmolarity, thus proportioning the cell resistance against saline stress (Gregory et al., 2019). Here, we found genes coding for ectoine synthesis in genomic islands, which are usually enriched in secondary metabolites genes, providing evidence that secondary metabolism is linked to functional adaptation (Penn 2009). Also, a siderophore cluster was only recognized in two strains, namely Cmf 13.9 and 96–373, consistent with what we observed in the genome browser.

Antiphage systems, whose variable possession in closely related strains, as it is our case, indicate high rate of horizontal gene transfer (Tesson et al., 2022). Those systems were identified for all the strains (Table 4), in a number from five to eight, as the average number for prokaryotic genomes which is 5 (Tesson et al., 2022). Among them, all the strains encode for RM and dGTPase, the most common antiphages systems together with Cas which, interestingly, is only present in the strains isolated from clams.

All the strains presented CRISPR sequences, in a variable number from 1 to 3 (Table 4). For the case of Cas cluster gene sequences, only the strains CMJ 9.4 and CMJ 9.11 presented 2 and 1, respectively. None intact prophage sequence was detected, although he majority of the strains showed 1 to 3 incomplete prophage sequences (Table 4). Only the environmental strain 96–376 did not present any prophage sequence, neither intact or incomplete or questionable.

Using CARD, four antibiotic resistance gene sequences were identified for all the strains (Table 4), coding for quinolone resistance protein QnrS2, two resistance-nodulation-cell division antibiotic efflux pumps (adeF and CRP) and a trimethoprim resistant dihydrofolate reductase dfrA6.

## Conclusions

The comparative genomic analysis of the *V. toranzoniae* strains revealed ample homology between them, with notable differences related to motility, capsule synthesis, iron acquisition system, or phage-related elements. The strains share a core genome of 2,953 genes out of a pangenome of 6,287 genes, according to GET_Homologues. Those strains grown in the same breeding area grouped phylogenetically together, thus the geographical conditions prevailing over the ecological ones. Finally, reclassification of R17 strain as *V. kanaloae* emphasizes the need for deposited sequences to be cured and properly designated, in order to avoid possible mistakes, especially among strains as similar as those belonging to the Splendidus clade within the genus *Vibrio*.

## Figures and Tables

**Table 1:** *Vibrio toranzoniae* strains included in the study.

Strain	Origin	Host	Date
CECT 7225^⊤^	Galicia, Spain	*Ruditapes decussatus*	2004
CMJ 9.4	Galicia, Spain	*Ruditapes philippinarum*	2005
CMJ 9.11	Galicia, Spain	*Ruditapes decussatus*	2005
Cmf 13.9	Galicia, Spain	*Ruditapes philippinarum*	2005
96–373	Valencia, Spain	Seawater	1996
96–376	Valencia, Spain	Seawater	1996

**Table 2:** Values of DDH among *V. toranzoniae* strains.

	CECT 7225^⊤^	CMJ 9.4	CMJ 9.11	Cmf 13.9	96–373	96–376	R17	R18	R19	*V kanaloae* CCUG 56968^⊤^
**CECT 7225^⊤^**	100.0									
**CMJ 9.4**	81.2	100.0								
**CMJ 9.11**	77.9	78.5	100.0							
**Cmf 13.9**	80.7	79.6	77.1	100.0						
**96–373**	82.3	80.9	77.5	80.30	100.0					
**96–376**	80.6	80.4	77.1	78.90	80.00	100.0				
**R17**	58.7	58.9	60.6	59.5	58.6	58.6	100.0			
**R18**	58.5	58.9	60.6	59.4	58.5	58.5	100.0	100.0		
**R19**	58.5	58.9	60.6	59.4	58.5	58.5	100.0	100.0	100.0	
**V. kanaloae** **CCUG 56968^⊤^**	61.6	62.0	61.0	62.5	61.8	61.8	86.4	86.3	86.3	100.0

**Table 3:** Genome statistics for *V. toranzoniae* strains.

	CECT 7225^⊤^	CMJ 9.4	CMJ 9.11	Cmf 13.9	96–373	96–376
Genome size (Mb)	4.61	4.76	4.56	4.71	4.64	4.64
G + C content	44	43.9	43.8	43.9	43.9	43.9
Number of contigs	2	299	311	192	132	135
Coding sequences	4164	4236	4053	4158	4258	3826
RNA genes	172	158	181	181	126	146

**Table 4:** Summary of genetic traits present in *V. toranzonaie* strains.

	CRISPR sequences	Incomplete prophage sequences	Secondary metabolites	Virulence factors	Phage defense elemente
CECT 7225^⊤^	1	1	PUFAs, ectoine, arylpolyene, bacteriocine, betalactone	Hemolysin, VirK, virulence-associated Efamily protein, Iron-regulated protein IrgB	RM (3), Druantia, Zorya, Cas, dGTPase, Viperin
CMJ 9.4	3	2	PUFAs, ectoine, arylpolyene, bacteriocine, betalactone	Hemolysin, Iron-regulated protein IrgB	RM (2), Cas (2), dGTPase, BstA
CMJ 9.11	1	3	PUFAs, ectoine, arylpolyene, bacteriocine, betalactone	Hemolysin, VirK	RM (2), dGTPase, BREX, DTR, Cas, Rst-sirtuin-like
Cmf 13.9	2	1	PUFAs, ectoine, arylpolyene, bacteriocine, betalactone, siderophore	Hemolysin, probable RTX,Iron-regulated protein IrgB, siderophore assembly kit	RM (2), dGTPase, Cas, Rst-sirtuin-like
96–373	1	1	PUFAs, ectoine, arylpolyene, bacteriocine, betalactone, siderophore	Hemolysin, Iron-regulated protein IrgB	RM (3), dGTPase, Septu, Hachiman
96–376	1	0	PUFAs, ectoine, arylpolyene, bacteriocine, betalactone	Hemolysin, Iron-regulated protein IrgB	RM (2), Nhi, Zorya, Kiwa, dGTPase, Rst-ATPase

All strains harbour the antimicrobial peptides adeF, CRP, QnrS2, drfA6

**Table 5:** Number of identified GIs in *V. toranzoniae* strains.

	CECT 7225^⊤^			CMJ 9.4			CMJ 9.11			Cmf 13.9			96–373			96–376		
	S	I	T	S	I	T	S	I	T	S	I	T	S	I	T	S	I	T
*V. anguillarum*	29	8	37	33	12	45	25	13	38	26	12	38	29	10	39	26	12	38
*V. splendidus*	27	9	36	34	10	44	28	15	43	26	9	35	30	12	42	26	10	36
*V. vulnificus*	30	9	39	34	9	43	26	14	40	25	10	35	28	12	40	27	10	37

S = SIGI-HMM method, I = IslandPath-DIMOB method, T = total.

## Data Availability

Sequence data that support the findings of this study have been deposited in GenBank under the accession numbers GCA-001541335.1 (V. toranzoniae CECT 7225T ), GCA-009906155.1 (V. toranzoniae 96–373), GCA-009906235.1 (V. toranzoniae 96–376), GCA-009906185.1 (V. toranzoniae CMJ 9.4), GCA-009906175.1 (V. toranzoniae CMJ 9.11), GCA-009906085.1 (V. toranzoniae Cmf 13.9) and GCA-001995825.2 (V. kanaloae R17).
